# Equivalent Circuit Models of the Piezoelectric Transducers for Medical Ultrasound Applications

**DOI:** 10.3390/s26196299

**Published:** 2026-10-05

**Authors:** Hojong Choi

**Affiliations:** Department of Electronic Engineering, Gachon University, 1342 Seongnam-daero, Sujeong-gu, Seongnam 13120, Republic of Korea; hojongch@gachon.ac.kr; Tel.: +82-31-750-5591

**Keywords:** equivalent circuit model, piezoelectric transducer, ultrasound system

## Abstract

Equivalent circuit models are crucial for estimating the performance of piezoelectric transducers. Certain techniques, such as electrical impedance matching between the transmitter and piezoelectric transducers, help optimize the performance of ultrasound systems. In addition, a straightforward model analysis is important for fabricating high-performance piezoelectric transducers. In this study, the presented Mason, Krimholtz–Leedom–Matthaei (KLM), and Butterworth–van Dyke (BVD) models and other modified equivalent circuit models used in piezoelectric transducers were analyzed with specific component parameters. In addition, specific parameters are provided when selecting certain equivalent circuit models. Therefore, ultrasound transducer and circuit designers can determine the type of equivalent circuit model to be used for enhancing the performance of the ultrasound transducer or system for medical ultrasound applications. The information provided by the equivalent circuit model could be helpful in fabricating mechanical piezoelectric transducers or designing electrical impedance-matching circuits in ultrasound systems.

## 1. Introduction

In medical ultrasound systems, the transmitter and receiver are electronic systems, and the transducers are mechanical devices [1,2]. In ultrasound systems, piezoelectric transducers are mechanical devices that are triggered by high-voltage signals from a transmitter and produce echo signals in the receiver [3,4]. In the transmitter, a power amplifier or pulse generator sends high-voltage signals to the piezoelectric transducers, which produce acoustic signals [5]. The received acoustic echo signals are converted into received electrical echo signals by the transducers [6]. Subsequently, the preamplifier and variable-gain or time-gain compensation amplifiers provide further amplification in the ultrasound receiver.

The magnitude and phase of the electrical impedances in the equivalent circuit models of the piezoelectric transducers vary with frequency [7]. Therefore, the equivalent circuit models for estimating the electrical impedance behavior are quite complicated; however, the frequency-dependent amplitudes can be estimated [8,9]. To achieve the high performance required of medical ultrasound systems, proper excitation from the power amplifier or pulse generator and adequate amplification from the preamplifier are essential [10,11]. Therefore, a proper analysis of the equivalent circuit models and transmitter/receiver electronics, even at the design level, is required. Academic ultrasound researchers, transducer fabrication designers, and circuit/system engineers must consider the electrical impedances of the piezoelectric transducers, based on the equivalent circuit model, to obtain proper performance in terms of sensitivity, bandwidth, and image resolution in ultrasound systems.

PMN-PT material exhibits a high piezoelectric strain coefficient (d_33_), electromechanical coupling coefficient (k_33_), and dielectric constant. However, due to their relatively low Curie and depoling temperatures, they are primarily used in intravascular or high-frequency imaging applications [12]. LiNbO_3_ material exhibits a high Curie temperature alongside a low dielectric constant; however, its lower d_33_ and k_33_ values relative to PMN-PT material limit its application to high-frequency eye imaging. PZN-PT material offers exceptionally high d_33_ and k_33_ values with high electromechanical coupling, but its lower thermal stability and potential for depolarization during fabrication restrict its use primarily to wideband medical ultrasound applications [12]. Compared to single crystals, lead zirconate titanate (PZT) ceramics such as PZT-5H remain widely used in medical ultrasound arrays due to their good electromechanical coupling coefficient, relatively high Curie temperature, excellent thermal stability, low fabrication cost, and mature manufacturing processes [13]. These material characteristics directly map to specific parameters in equivalent circuit models. Dielectric permittivity dictates the clamped capacitance, and the piezoelectric coefficient and elastic constants determine the electromechanical coupling coefficients [13]. The density and elastic constants govern resonance characteristics. Dielectric and mechanical losses contribute to the resistive components in the equivalent circuit model.

Compared with capacitive micromachined ultrasonic transducers, piezoelectric transducers do not require a high-voltage direct current (DC), and their sensitivity and efficiency are typically high [14,15]. Therefore, piezoelectric transducers are widely used in various medical ultrasound applications, such as medical imaging, cell trapping, brain stimulation and imaging, and therapy [16,17].

The equivalent circuit model is useful for estimating the performance of a piezoelectric transducer before transducer fabrication and for creating an impedance-matching network for the ultrasound system [18,19]. The finite element method (FEM) can produce highly accurate impedance values from a piezoelectric transducer because the FEM model can be applied to obtain the vibrational modes and mechanical structures [20]. The FEM can provide a dynamic behavior analysis of different structures with nodes [8]. Based on the FEM, commercial piezoelectric transducer software such as PZFlex (Weidlinger Associates Inc., New York, NY, USA) and commercial design software such as Multiphysics (COMSOL Inc., Burlington, MA, USA) and Workbench (ANSYS Inc., Canonsburg, PA, USA) have been used [21]. However, FEM can be time-consuming and is not straightforward for analyzing the performance of piezoelectric transducers [22]. Compared with FEM, the accuracy of the equivalent circuit model is typically lower, although the computation time is shorter for parameter selection [23]. Therefore, equivalent circuit models have been presented.

The Mason, Krimholtz–Leedom–Matthaei (KLM), and Butterworth–Van Dyke (BVD) models are representative examples of currently presented equivalent circuit models [24,25,26]. The Mason and KLM equivalent circuit models are useful for estimating the transducer performance before fabrication. The BVD model can be obtained from simulated FEM data or measured electrical impedances without mechanical information. The Mason model is directly derived from the characteristics of piezoelectric materials, such as acoustic (elastic and piezoelectric) and piezoelectric (dielectric) losses. In the Mason model, an ideal transformer is used to describe the relationship between the mechanical properties of piezoelectric materials using acoustic and electrical ports. The KLM model is a combined structure of an ideal transformer and a transmission line and is useful for multilayer structures such as matching layers. The KLM and Mason models describe the mechanical and electrical properties of piezoelectric transducers. These models have some limitations, such as a negative clamped capacitance and frequency-dependent ideal transformers. Commercial software, such as PiezoCAD (Sonic Concepts, Woodinville, WA, USA), has been used to design piezoelectric transducers before fabrication based on the KLM model [27]. The BVD model contains a resistor, capacitor, and inductor; therefore, it can be used in parallel structures to describe multiple resonant and anti-resonant frequency behaviors. However, it is difficult to use this model for transducer fabrication without detailed mechanical components.

This review article is organized into five sections. Section 1 provides a brief description of the equivalent circuit models. Section 2 describes the fundamental equivalent circuit models of Mason, KLM, and BVD and explains how they are used with specific components. Section 3 describes previously published equivalent circuit models of piezoelectric transducers used for medical ultrasound applications. Section 4 discusses the previously published equivalent circuit models. From Section 4, academic researchers and circuit and system engineers can understand how to utilize the equivalent circuit model for their specific applications. Finally, Section 5 presents the conclusions of this study.

## 2. Fundamental Concept of the Equivalent Circuit Models

**Figure 1:**
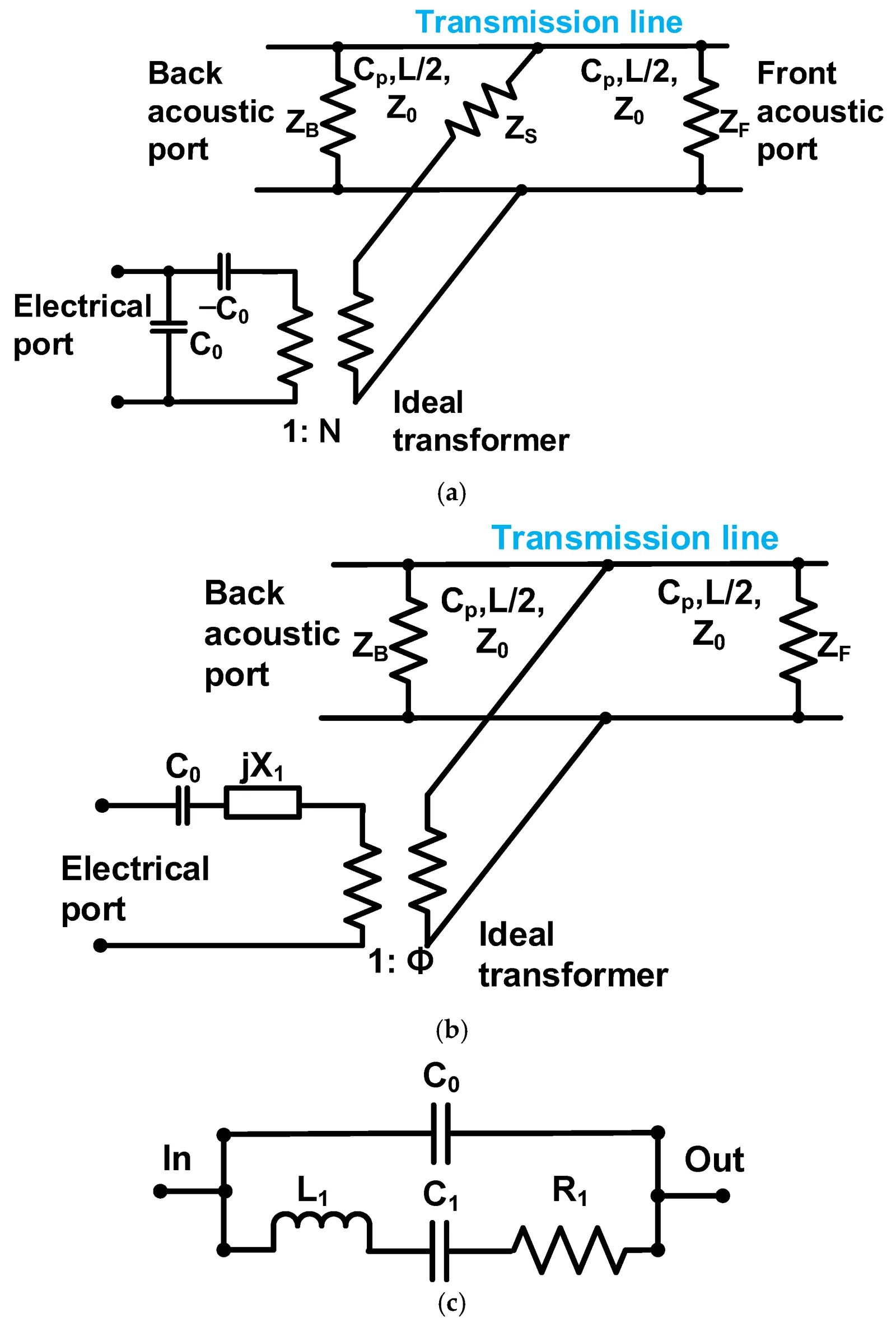
(**a**) Mason, (**b**) KLM, and (**c**) BVD equivalent circuit models. Redrawn based on Ref. [13].

Figure 1 shows schematics of the original Mason, KLM, and BVD models used to analyze the operating mechanism.

In the Mason model (Figure 1a), the transmission line represents the mechanical part of the piezoelectric transducer with back and front acoustic ports. Z_B_ and Z_F_ are the acoustic impedances at the back and front acoustic ports, respectively. Z_s_ is the impedance of the interconnection, where N is the turns ratio of the ideal transformer. L is the length of the transmission line in the direction of the acoustic waveform.

In the Mason model, a clamped capacitance (C_0_) and an ideal transformer represent the electromechanical conversion and attenuation between the mechanical and electrical ports. C_0_ and N can be calculated using Equation (1) [28]:

(1) $$C_{0}=\varepsilon_{0}\varepsilon_{r}\left(\frac{A}{d}\right),\;N=h_{33}C_{0}$$

where A and d are the area and thickness of the piezoelectric material, respectively; ε_0_ and ε_r_ are the vacuum permittivity and relative dielectric constant of the piezoelectric material, respectively; and h_33_ is the piezoelectric transmitting constant.

The acoustic impedances (Z_0_ and Z_S_) are expressed by Equation (2) [28]:

(2) $$Z_{0}=\rho Av_{D},\;Z_{S}=jZ_{0}\tan(\frac{\omega t}{2v_{D}})$$

where ρ and v_D_ are the density of the medium and longitudinal acoustic velocity, respectively, while t and v_D_ are the thickness and longitudinal velocity within the piezoelectric medium.

In the KLM model (Figure 1b), an ideal transformer with capacitive and reactive components at the electrical port models the electrical behavior of the piezoelectric transducer. C_p_ is the acoustic velocity of the piezoelectric material. In the transmission line model, impedance Z_o_ is the acoustic impedance of the piezoelectric material. In Equation (3), Φ is the ideal electromechanical transformer ratio defined as [11]

(3) $$\Phi =k{\left(\frac{\pi }{w_{0}C_{0}Z_{0}}\right)}^{\frac{1}{2}}\frac{\sin\frac{w}{2w_{0}}}{\frac{w}{2w_{0}}},$$

where k is the electromechanical coupling coefficient governing the energy conversion between electrical and mechanical domains, w_0_ is the angular anti-resonant frequency, and w is the operating frequency of the piezoelectric material.

In the KLM model, the piezoelectric material properties, such as backing and matching layers, together with the piezoelectric medium, directly dictate the resonance and anti-resonant characteristics. In Equation (4), X_1_ is the electrical impedance owing to the acoustic transmission line and is given by

(4) $$X_{1}=\frac{-C_{0}}{{k_{t}}^{2}(\sin\frac{\omega }{\omega_{0}})}$$

In the BVD model (Figure 1c), the clamped capacitance (C_0_), which represents the intrinsic dielectric capacitance of the piezoelectric layer, can be derived using Equation (5) [29,30].

(5) $$C_{0}=\frac{\left|\frac{X_{\mathrm{res}}}{\sqrt{{R_{\mathrm{res}}}^{2}+{X_{\mathrm{res}}}^{2}}}\right|}{w_{\mathrm{res}}},$$

where R_res_ and X_res_ are the resistance and admittance at the angular resonant frequency (w_res_), respectively.

The resistance (R_1_) represents the loss at the resonant frequency, and the capacitance (C_1_) can be obtained from Equation (6) [31,32]:

(6) $$C_{1}=C_{0}\left({\left(\frac{w_{\mathrm{anti}}}{w_{\mathrm{res}}}\right)}^{2}-1\right),$$

where w_anti_ is the angular anti-resonant frequency.

The inductance (L_1_) can be obtained using Equation (7) [33]:

(7) $$L_{1}=\frac{1}{{w_{\mathrm{res}}}^{2}C_{1}}$$

In the BVD model, R_1_ represents the mechanical damping or energy loss in the electrical equivalent component [21]. L_1_ and C_1_ are related to the elastic stiffness, density, and thickness of the piezoelectric material [18], while k_t_ is related to the energy conversion between the mechanical and electrical ports. These elements show the mechanical properties of the piezoelectric material at the resonance and anti-resonance frequencies. Unlike the Mason and KLM models, the BVD model does not maintain a direct one-to-one relationship with material properties; instead, its electrical components are extracted from the measured impedances around the resonance and anti-resonance frequencies, offering a compact impedance behavior for circuit-level analysis. Conversely, the Mason and KLM models are better suited when the material properties and acoustic layer configurations need to be explicitly modeled.

The currently used representative equivalent circuit models are based on the Mason, KLM, and BVD models, except for network models. Section 3 describes the analysis of the currently presented models and component parameters for specific medical ultrasound applications.

## 3. Analysis of the Presented Equivalent Circuit Models

Previously published articles used different symbols and circuit schematics to represent the components of the equivalent circuit models of piezoelectric materials for certain medical ultrasound applications and to explain specific behaviors. However, identical symbols such as impedance (Z), length (L), the electro-mechanical transformer ratio (ϕ), clamped capacitance (C_0_), the cross-sectional area (A), the electro-mechanical coupling factor (k_t_), the thickness of the piezoelectric material (T), and the force (F) in the acoustic ports have been used to help readers to easily differentiate between the characteristics of the currently introduced equivalent circuit models. In addition, several equivalent circuit models were redrawn using the same symbols and circuit schematics so that the different components and circuit schematics could be easily recognized.

**Figure 2:**
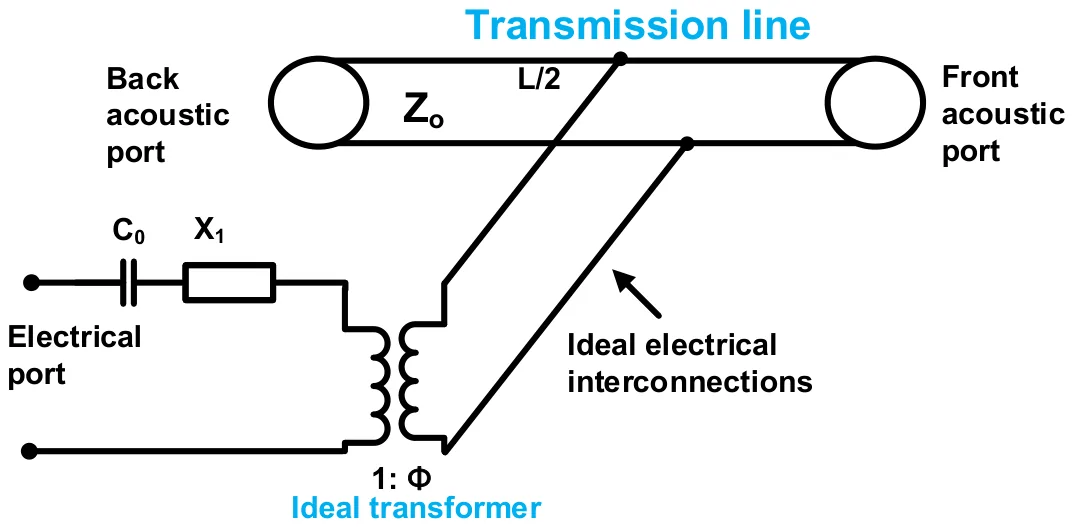
The equivalent circuit model was presented by Leedom et al. and reproduced from Ref. [34]. Copyright 1971, IEEE.

Figure 2 shows the equivalent circuit model introduced by Leedom et al. [34]. The original KLM model proposed by Krimholtz, Leedom, and Matthaei introduced a transmission line representation of acoustic propagation as an alternative formulation to Mason’s model for one-dimensional wave analysis. Leedom et al. extended this framework to transducers with arbitrary symmetry and side-electrode configurations.

The transmission line represents the mechanical part of the piezoelectric transducer with the back and front acoustic ports. In the transmission line, impedance Z_o_ is the acoustic impedance of the piezoelectric material. C_0_ is the clamped capacitance. L is the length of the transmission line in the direction of the acoustic waveform and is equal to the length of the piezoelectric transducer. An ideal transformer with a capacitor (C_0_), reactance (X_1_), and electrical port represents the electrical component of the piezoelectric transducer. Φ is the ideal mechanical–electrical transformer ratio (ϕ), which is described by

(8) $$\Phi =\frac{\alpha \beta c_{33}^{D}D_{0}}{P(k)},$$

where α and β are the attenuation coefficient and wavenumber of the piezoelectric material, respectively; C_33_^D^ is the elastic stiffness for constant electric displacement; D_0_ is the electric displacement; and p(k) is an even function if p(x) = 0 in the range −l/2 < x < l/2.

For the thickness mode of a piezoelectric material, the piezoelectric excitation is defined as p(x) = h_33_(x)D_3_(x).

(9) $$C_{0}=\frac{\alpha \beta D_{0}}{\left(\int_{-\infty }^{+\infty }D_{3}(x)\left\{\beta_{33}^{s}\left(x\right)-\frac{h_{33}^{2}\left(x\right)}{c_{33}^{D}}\right\}\mathrm{dx}\right)},$$

(10) $$X_{1}={\left[\left(2c_{33}^{D}v\alpha \beta D_{0}^{2})H({p\left(k\right)}^{2}\right)\right]}^{-1},$$

where D_3_(x) is the electric displacement, which is the reference for the phase quantities; β_33_^S^ is the dielectric impermeability; and v is the acoustic velocity.

**Figure 3:**
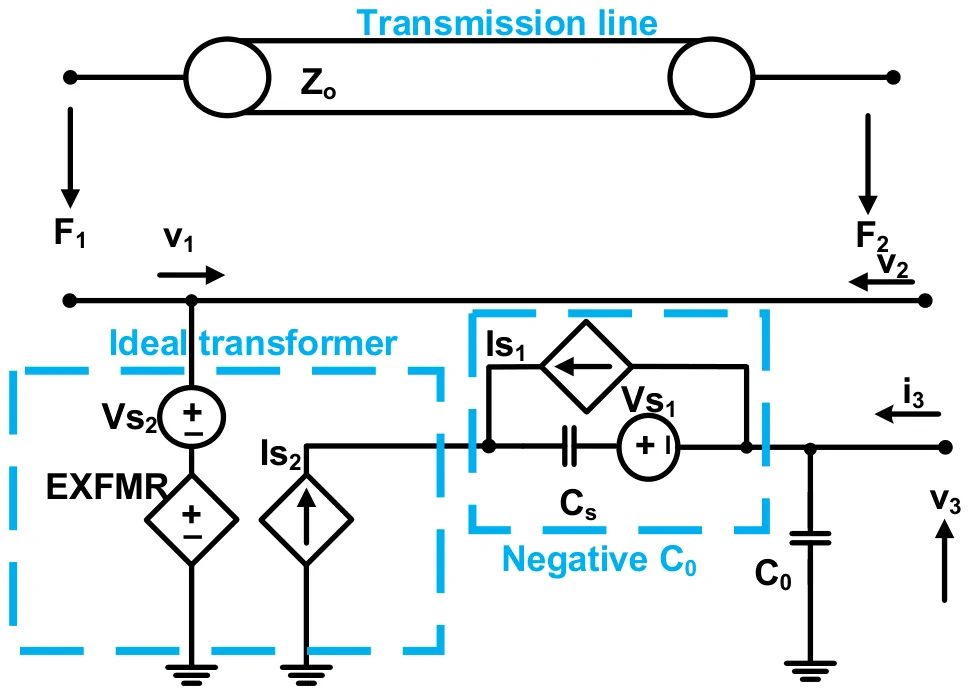
The equivalent circuit model was presented by S.A. Morris et al. Redrawn based on Ref. [35]. Copyright 1986, IEEE.

Figure 3 shows the modified Mason equivalent circuit. The equivalent-circuit model is useful for simulating a transducer in the SPICE program [35].

The ideal transformer was converted into a source voltage model because the negative capacitance value could not be used in the SPICE program [36]. Thus, the negative-clamped capacitance (C_0_) also changed the capacitance (C_s_) and voltage and current sources (V_s1_ and I_S1_) used in the SPICE program. F_1_ and F_2_ are forces on the front and back load sides, respectively. V_3_ and I_3_ are the variables in the electrical ports, respectively. V_S1_ and V_S2_ are the dependent voltage sources of I_S1_ and I_S2,_ which are the current sources, respectively. Z_o_ and C_0_ are the acoustic impedance and clamped capacitance of the transmission line and piezoelectric material, respectively.

**Figure 4:**
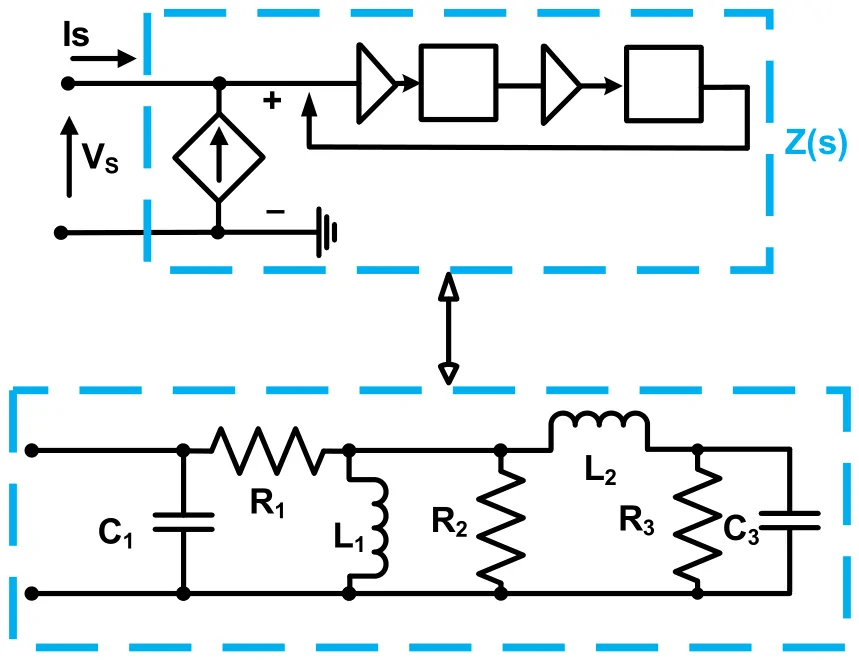
Analytical model (**top**) and equivalent circuit model (**bottom**). Redrawn based on Ref. [37]. Copyright 1993, IEEE.

Figure 4 shows a black-box model of the equivalent circuit used to create an analytical model in the Laplace domain [37]. This model does not require information regarding the piezoelectric material specifications. This functional black-box model Z(s) was used in an analog circuit simulator. An analytical model was applied using a trial-and-error method. L_1_, L_2_, C_1_ to C_3_, and R_1_ to R_3_ represent the equivalent inductive, capacitive, and resistive components to describe the capacitive behaviors of the impedances in the piezoelectric materials.

In Figure 4, Z(s) represents the input impedance of the ultrasonic transducer. V_S_ and I_S_ are the probe voltage and current, respectively. In the top diagram, the triangular and square functional blocks represent the amplifier and filter, respectively [37]. The bottom diagram shows the equivalent circuit of the transducer. The analytical model was obtained from the measured impedance and acoustic echo signal sampling data obtained using a hydrophone. Therefore, this equivalent circuit model can be used to match the impedances of the front-end electronics.

**Figure 5:**
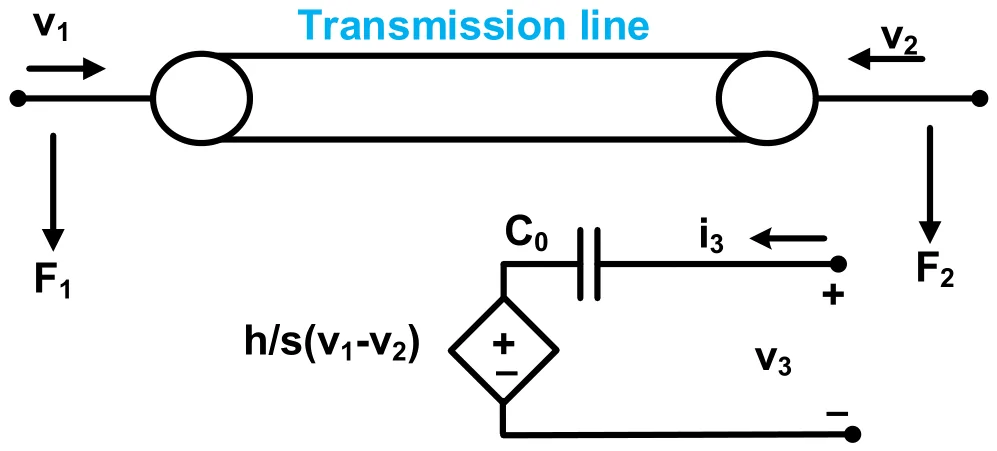
The equivalent circuit model was introduced using a controlled source. Reproduced based on Ref. [38]. Copyright 1994, IEEE.

Figure 5 shows the equivalent circuit model using controlled sources for the SPICE program proposed by Leach et al. Instead of a transformer, which shows the coupling between the electrical and mechanical parts, a controlled source was used for the thickness-mode transducer. In the Mason model, the transformer generates the negative clamp capacitance.

The top (transmission line) and bottom (controlled source) parts represent the mechanical and electrical parts, respectively. C is the piezoelectric constant, and s is the Laplace operator. v_1_ and v_2_ denote the acoustic forces at the back and front acoustic ports, respectively. v_3_ and i_3_ are the dependent voltage and current sources of the electrical component, respectively. The controlled source in the h/s(v_1_ − v_2_) term of the mechanical part shows the common-mode voltage at two points on the transmission line. Instead of a transformer, Leach et al. used a controlled source for flexible selection of analog circuits. This is the equivalent circuit model used to replace the ideal transformer and represents the coupled relationship between the mechanical and electrical ports. Therefore, the negative-clamped capacitance was removed.

**Figure 6:**
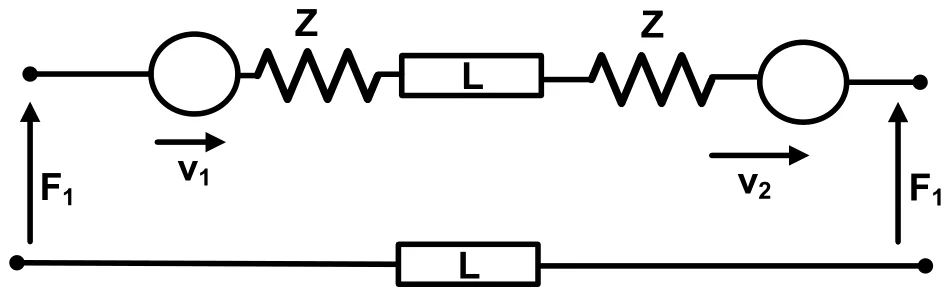
The transmission line model of the equivalent circuit. Redrawn based on Ref. [39]. Copyright 1996, IOP Science.

Figure 6 shows a new transmission line model for the equivalent circuit model of a multilayered piezoelectric transducer presented by Chubachi et al. [39]. This transmission line was used to analyze the effects of the electrical terminal load caused by velocity distribution. Therefore, the electrical impedances in the frequency-dependent behavior can be estimated. Z represents the variable impedance in the equivalent circuit model. L is the length of the transmission line.

**Figure 7:**
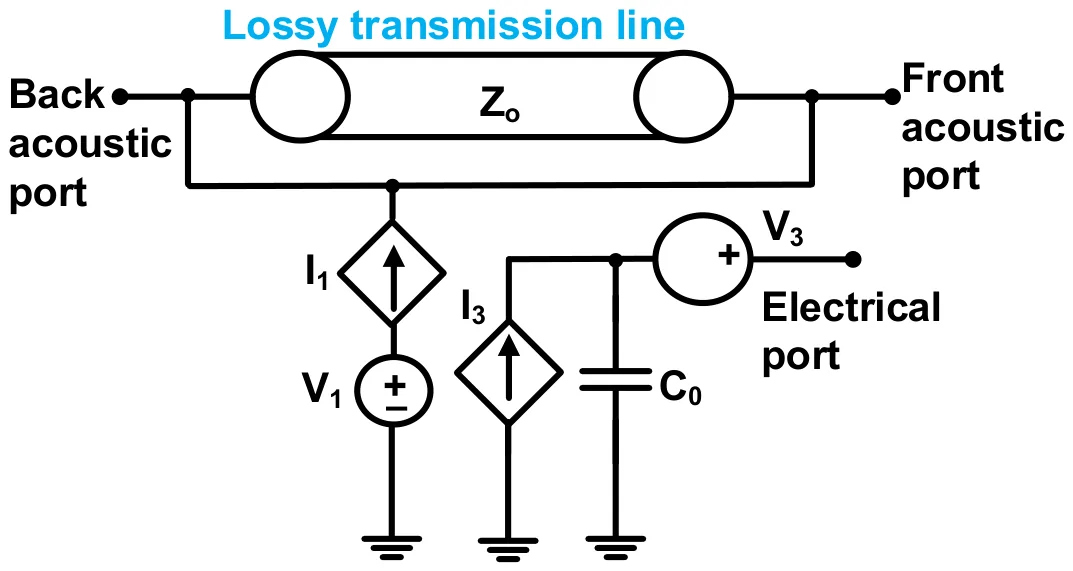
The transmission line model of the equivalent circuit. Adapted with permission from Ref. [40]. Copyright 1997, IEEE.

Figure 7 shows the equivalent circuit model using a lossy transmission line introduced by Püttmer et al. The mechanical loss can be modeled using a lossy transmission line [40].

C_0_ is the clamped capacitance, V_1_ and V_3_ are voltage sources, and I_1_ and I_3_ are current sources. Equations (11) and (12) show the controlled voltage source (I_1_) and characteristic impedance of the lossy transmission line, respectively [30]:

(11) $$V_{1}=C_{0}\frac{\mathrm{dI}(V_{1})}{\mathrm{dt}},$$

Z_0_ = Aρv_p_, (12)

where ρ is the density, v_p_ is the acoustic velocity, and A is the cross-sectional area of the piezoelectric material.

**Figure 8:**
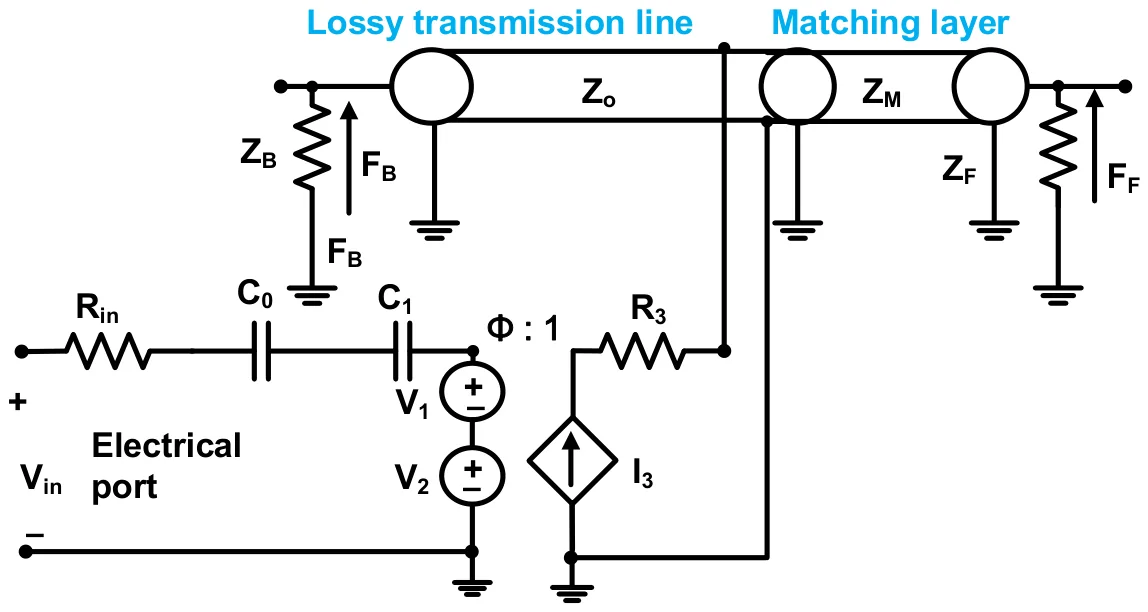
The modified KLM equivalent circuit model with lossy transmission line and matching layer. Redrawn based on Ref. [41]. Copyright 1999, Elsevier.

Figure 8 shows the modified KLM equivalent circuit model introduced by Wu et al. [41]. This model was proposed to analyze a thickness-mode piezoelectric material with a lossy transmission line and matching layer so that it could be simulated using the P-SPICE program. This model was intended for a piezoelectric transducer with low loss and wide bandwidth, which is used in medical ultrasound systems. A wide bandwidth and low sensitivity are preferred for medical imaging applications [42]. The mechanical part of the piezoelectric material comprises the lossy transmission line and matching layer.

R_in_ and R_3_ are internal parasitic primary and secondary resistances in the transformer. Z_0_ and Z_M_ represent the acoustic impedances of the lossy transmission line and matching layer, respectively. Z_B_ and Z_F_ are the acoustic impedances of the back and front acoustic ports, respectively. F_B_ and F_F_ denote the acoustic forces acting on the back and front acoustic ports. In addition to the mechanical part, there is an electrical component of the piezoelectric material. In the electrical part, V_1_ and E_1_ represent the primary voltages of the transformer. I_3_ represents the secondary current of the transformer. C_0_ and C_1_ represent the clamped and second-term capacitances, respectively, as shown in Equations (13) and (14) [41]:

(13) $$C_{0}=\frac{2\varepsilon_{33}^{S}A}{T},$$

(14) $$C_{1}=-\frac{C_{0}}{k_{T}^{2}}\frac{1}{\mathrm{sinc}(\frac{\mathrm{Tw}}{\pi V_{c}})},$$

where ε_33_^S^ is the dielectric constant. A, k_T_, and T are the cross-sectional area, electromechanical coupling factor, and thickness of the piezoelectric material, respectively.

In Equation (15), Φ represents the transformer ratio of the impedance of the electrical part to the mechanical part.

(15) $$\Phi =\frac{2h_{33}\sin\frac{T}{{2V}_{C}}}{Z_{C}w},$$

where h_33_, V_c_, Z_c_, and w denote the piezoelectric coefficient, sound velocity, acoustic impedance, and angular frequency, respectively.

**Figure 9:**
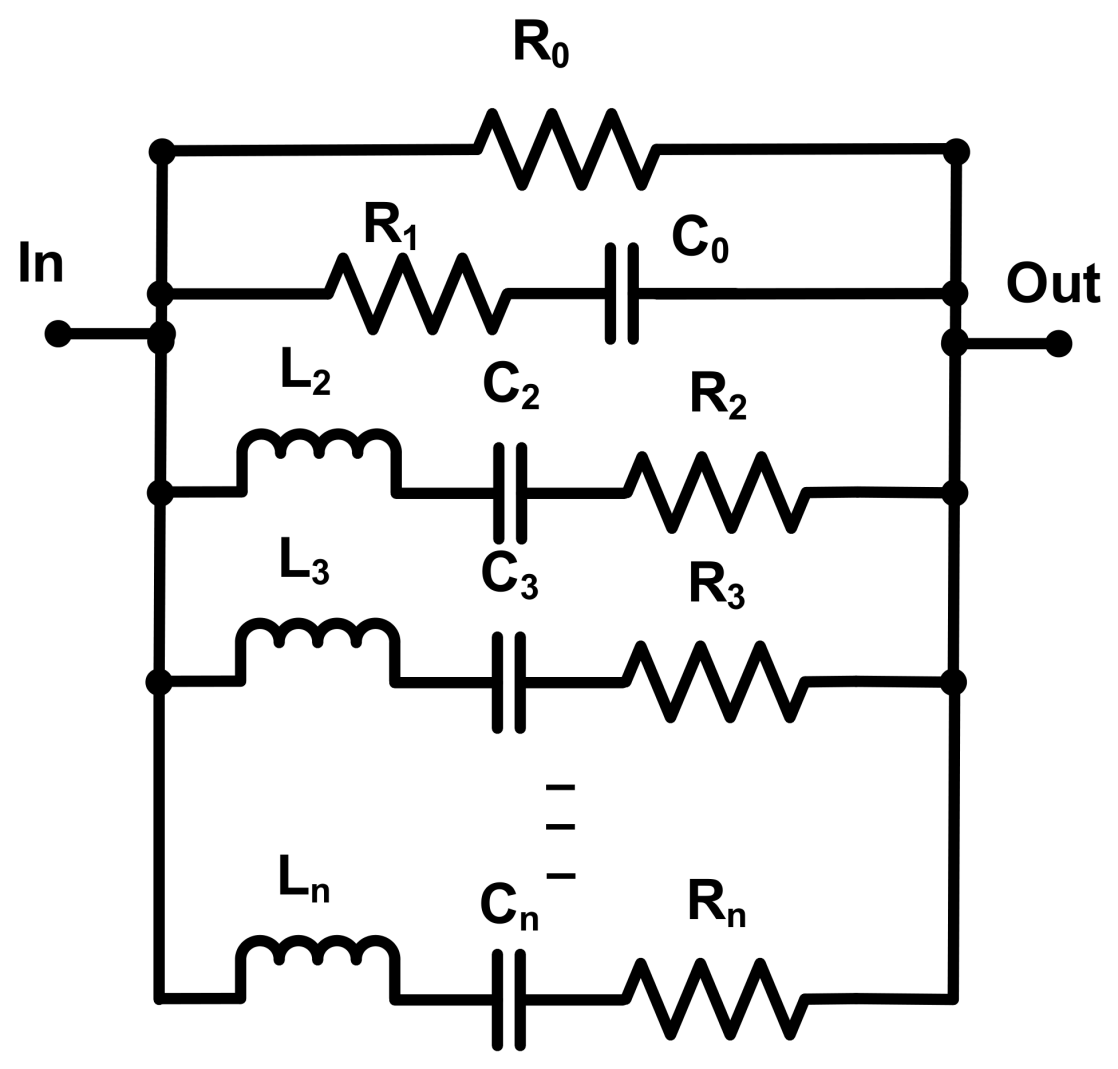
The BVD equivalent circuit model was introduced by Guan et al. Redrawn from Ref. [43]. Copyright 2004, IEEE.

Figure 9 shows the equivalent circuit model used to improve the accuracy of the BVD model, which deteriorated because of energy dissipation. This is because the BVD model cannot estimate the properties of piezoelectric materials with losses [43]. C_0_ is the clamped capacitance. The values of resistances (R_0_, R_1_, R_2_, R_3_, and R_n_) can be determined from the value of energy dissipation [43]. The resistors (R_2_, R_3_, ∙∙∙ R_n_), inductors (L_2_, L_3_, ∙∙∙ L_n_), and capacitors (C_2_, C_3_, ∙∙∙ C_n_) correspond to the effective mass, damping coefficient, and compliance (spring constant) of the piezoelectric material, respectively [43]. The resistors (R_0_ and R_1_) represent charge leakage and dielectric energy losses [43]. The series resistor–inductor–capacitor (RLC) structures represent the multiple resonance and anti-resonant modes. Thus, this equivalent circuit is particularly suitable for modeling transducers operating across wide frequency bands with multi-mode resonances. To obtain each resistance, capacitance, and inductance value, each resonance point needs to be derived from the measured resonance points.

**Figure 10:**
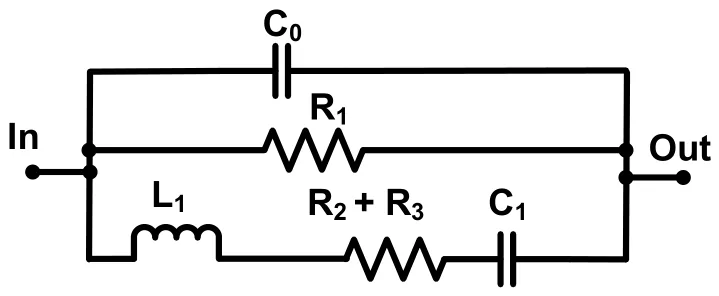
The modified BVD equivalent circuit model. Reproduced from Ref. [44].

Figure 10 shows the modified BVD equivalent circuit model presented by Fattah et al. This model was introduced to account for higher-order harmonic vibration modes over a broad bandwidth [44]. The inductive component models the mechanical damping factor in PZT-5H piezoceramic materials. The resistors (R_1_, R_2_, and R_3_) represent electric-field-dependent dielectric loss, strain-dependent mechanical loss, and acoustic radiation resistance loaded by the medium, respectively [44].

A modified BVD model was derived to account for harmonic components. Q_m_ represents the quality factor of the acoustic impedance. R_1_ and Q_m_ are expressed as shown in Equations (16) and (17) [45,46].

(16) $$R_{1}=\frac{1}{Q_{m}C_{0}w_{s}},$$

(17) $$Q_{m}=\frac{\sqrt{L_{1}C_{1}}}{R_{1}},$$

where C_0_ is the clamped capacitance, and ω_s_ is the angular resonant frequency

**Figure 11:**
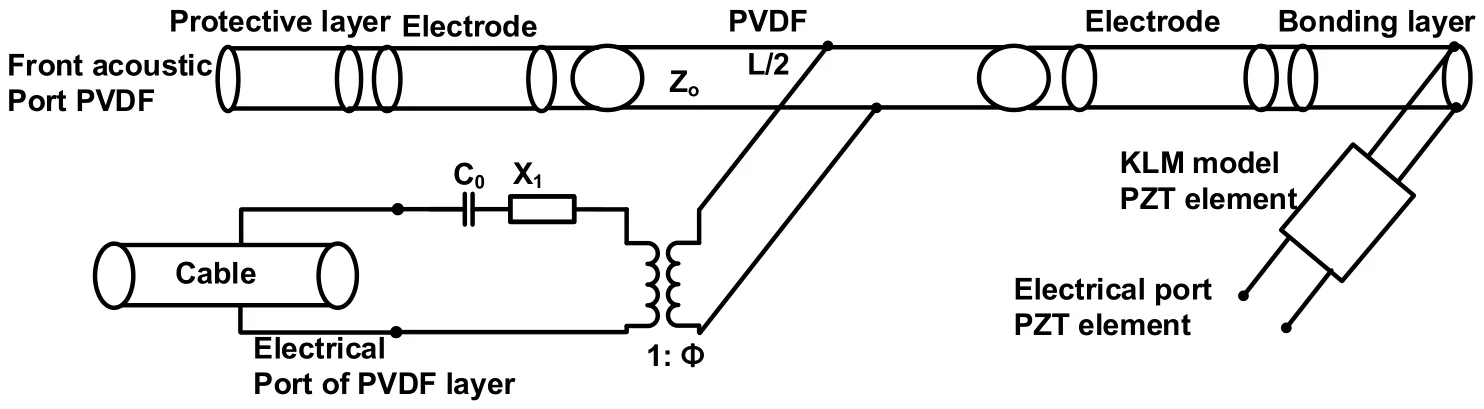
The KLM equivalent circuit model for the piezoelectric transducer has a multilayer structure. Adapted with permission from Ref. [47]. Copyright 2006, Elsevier.

Figure 11 shows the KLM equivalent circuit model of the piezoelectric transducer for acoustic bladder volume measurement. The piezoelectric transducer is composed of lead zirconate titanate (PZT) material for transmission and a polyvinylidene fluoride (PVDF) layer for reception [47]. The presented piezoelectric transducer has a multilayer structure; therefore, the equivalent circuit model includes the bonding layer, electrodes, PZT element, protective coating, and PVDF layer, as shown in Figure 11. To simplify the analysis, the PZT element in the KLM model is represented as a box [47].

**Figure 12:**
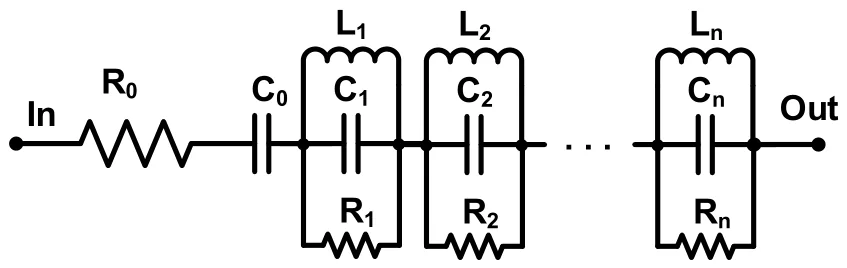
The equivalent circuit model was proposed by Kim et al. and redrawn from Ref. [49]. Copyright 2008, IEEE.

Figure 12 shows the equivalent circuit model introduced by Kim et al. The multilayer structures of piezoelectric materials can have multiple resonance frequencies. In addition, a medium load changes the electrical impedance [48]. They concluded that the modified BVD model was a simple model. The values of the inductances (L_1_, L_2_, … L_n_), capacitances (C_1_, C_2_, … C_n_), and resistances (R_1_, R_2_, … R_n_) describe the multiple resonances of the PZT material structures, which were obtained from the measured impedances of the PZT materials [49]. The resistance (R_0_) and capacitance (C_0_) were obtained from the measured impedance of the loaded PZT material.

**Figure 13:**
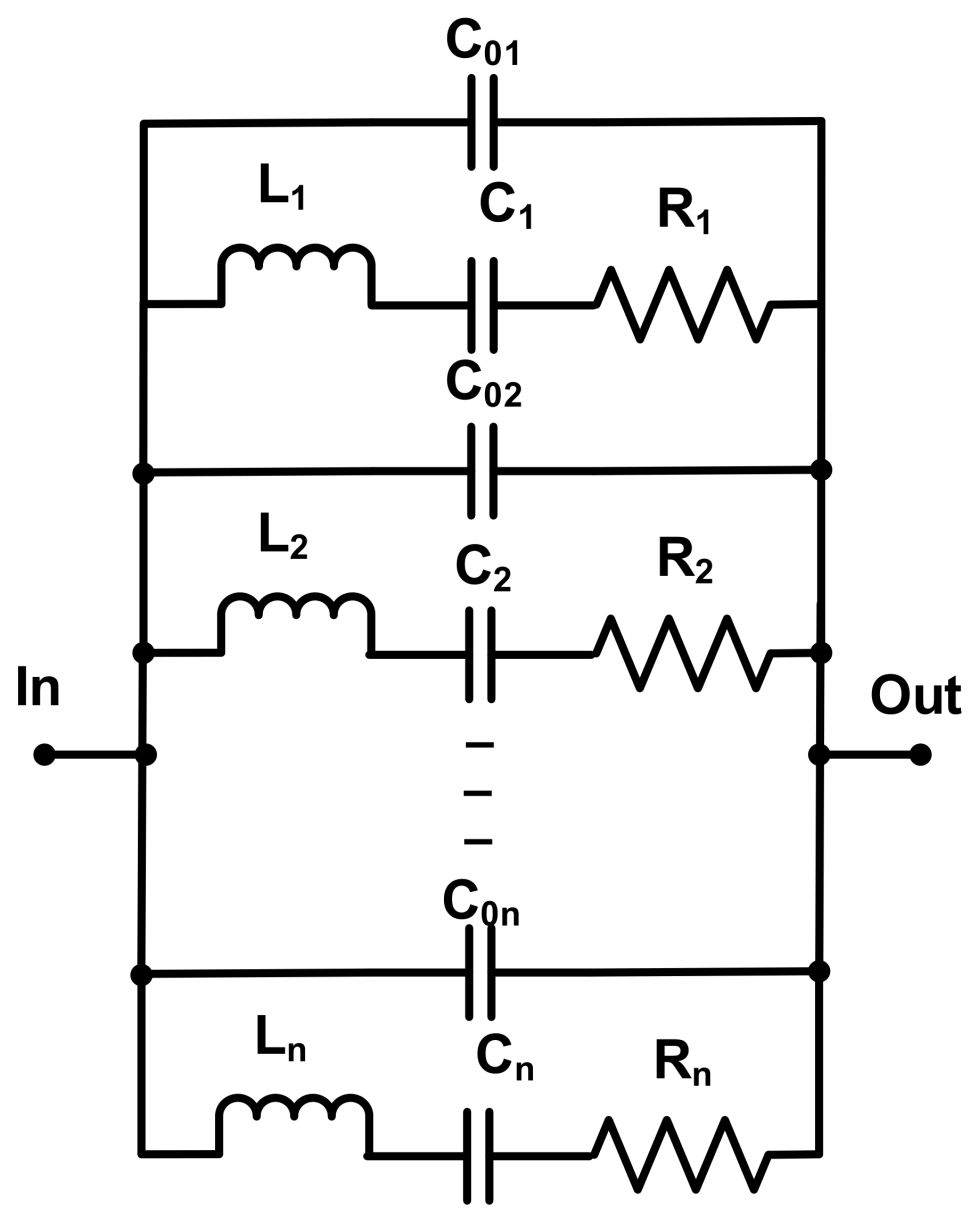
The equivalent circuit model. Redrawn from Ref. [50]. Copyright 2011, IEEE.

Figure 13 shows the equivalent circuit model for broadband impedance matching of piezoelectric transducers. The proposed model supports multiple BVD models with multiple resonant frequencies [50]. To obtain the values of capacitances and inductances, the measured admittance was obtained using an impedance analyzer. As shown in Figure 13, the parallel connection of multiple BVD models can produce multiple resonance and anti-resonance points. Therefore, the admittance values at the series and parallel resonances have the maximum and minimum values, respectively.

The magnitude of the admittance at the series resonance point should be the largest; therefore, the imaginary part of Equation (18) must be zero. Therefore, the resistance (R_1_) can be obtained using Equation (19) [51]. w_s1_ and w_p1_ are the first angular resonant and anti-resonant frequencies, respectively. Capacitance (C_01_) can be obtained using Equation (20). Equations (18)–(20) are defined as follows:

(18) $$Y_{1}=\frac{1}{R_{1}}+jw_{S1}C_{01},$$

(19) R1=ReY1(wS1)−1,

C_01_ = Im[Y_1_(w_S1_)]. (20)

The admittance at the parallel resonance point is the smallest; therefore, the real part of the admittance is zero in Equation (21) [52]. Therefore, inductance (L_1_) can be calculated using Equation (22) [53]:

(21) $$C_{1}=C_{01}\left\{{\left(\frac{w_{P1}}{w_{S1}}\right)}^{2}-1\right\},$$

(22) $$L_{1}={\left[C_{1}{\left(w_{S1}\right)}^{2}\right]}^{-1}.$$

**Figure 14:**
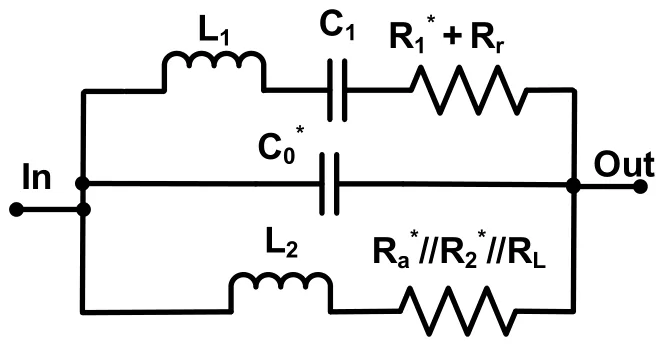
The equivalent circuit model. Redrawn from Ref. [54]. Copyright 2012, IEEE.

Figure 14 shows the modified BVD model derived by Choi et al. for high-frequency ultrasound imaging applications. In the model, each component value was obtained from the measured impedance magnitude and phase graphs [54]. The resistance (R_L_) was assumed to be a negligible loss term. This model provides both mechanical and electrical losses.

The values of resistance (R_1_*) and inductance (L_1_) can be obtained using Equations (23) and (24), respectively [54]:

(23) $${R_{1}}^{*}=R_{1}\cdot \cos(\theta_{\mathrm{res}}),$$

(24) $$L_{1}={\left[w_{\mathrm{res}}C_{1}\right]}^{-1},$$

where w_res_ and θ_res_ are the angular resonant frequency and phase angle, respectively.

The clamped capacitance (C_0_*) and secondary capacitance (C_1_) can be obtained from Equations (25), (26) and (27), respectively [54]:

(25) $${C_{0}}^{*}=C_{0}-\frac{\left(C_{1}//C_{0}\right)}{2Q_{\mathrm{anti}}}\;\mathrm{if}\;Q_{\mathrm{anti}}\;\mathrm{is}\;\mathrm{high},$$

(26) $${C_{0}}^{*}=C_{0}+\frac{\left(C_{1}//C_{0}\right)}{2Q_{\mathrm{anti}}}\;\mathrm{if}\;Q_{\mathrm{anti}}\;\mathrm{is}\;\mathrm{low},$$

(27) $$C_{1}=\left|C_{0}\cdot ({\left(\frac{w_{\mathrm{anti}}}{w_{\mathrm{res}}}\right)}^{2}-1)\right|,$$

where w_anti_ is the angular anti-resonant frequencies. Q_anti_ is the quality factor of the anti-resonant frequency. As shown in Equations (28) and (29), the resistance values (R_r_ and R_a_) were obtained from the piezoelectric transducer data [54]:

(28) $$R_{r}=\frac{\pi (Z_{1}+Z_{2})}{4k_{t}^{2}w_{\mathrm{res}}C_{0}Z_{C}}$$

(29) Ra*=Re4kt2ZCC0(Z1+Z2)3C02wanti,

where Z_1_ and Z_2_ denote the acoustic impedances at the front and back acoustic ports, respectively. k_t_ is the electromechanical coupling factor, and Z_c_ is the acoustic impedance.

The inductance (L_2_) and real resistance (R_2_*) were obtained using Equations (30) and (31), respectively [54]:

(30) $$L_{2}=\frac{R_{2}\cdot Q_{\mathrm{anti}}}{w_{\mathrm{res}}}$$

(31) $${R_{2}}^{*}=R_{2}\cdot \cos(\theta_{\mathrm{anti}}),$$

where R_2_ and θ_anti_ are the resistance and phase angle at the anti-resonant frequency, respectively.

In the impedance graph, the magnitudes and phases were compared among a 6 MHz PZT-5H transducer, the original BVD model, and the modified BVD models. At the anti-resonant frequencies, the magnitudes for the transducer, the original BVD model, and the modified BVD model were 91.4 Ω, 59.8 Ω, and 119.6 Ω, respectively [54]. The corresponding phase values were 2.2°, −38.4°, and 2.9°, respectively. Consequently, the modified BVD model demonstrated a superior ability to reduce the electrical impedance errors [54]. Consequently, the modified BVD model demonstrated a superior ability to reduce the electrical impedance errors.

**Figure 15:**
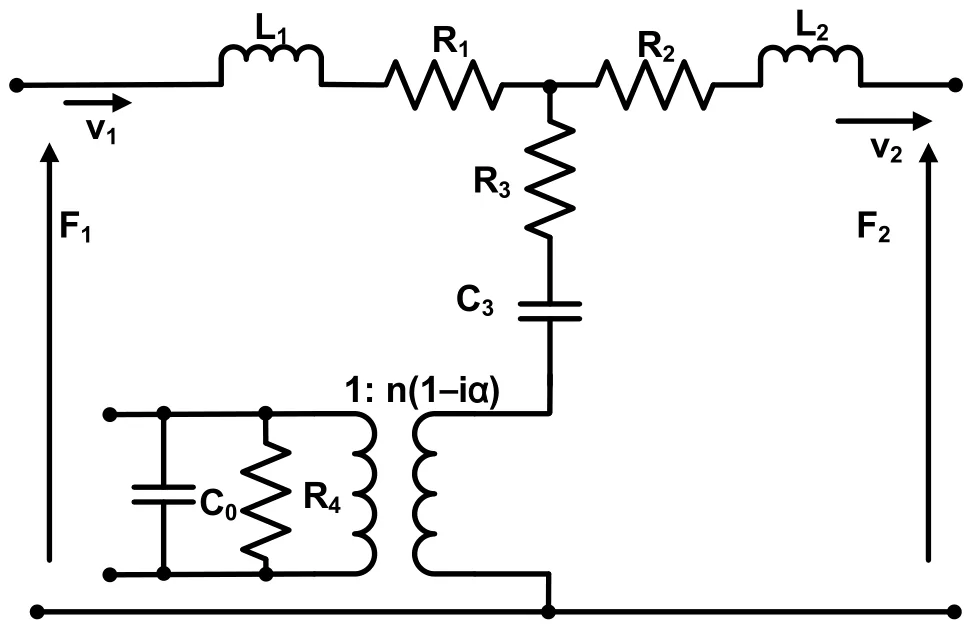
The modified Mason model. Redrawn from Ref. [55]. Copyright 2017, Elsevier.

Figure 15 shows an equivalent circuit based on the Mason model introduced by Dong et al. [55]. F1 and F2 are the forces acting on the back and front acoustic ports, respectively. The Mason model contains three types of losses: dielectric, elastic, and piezoelectric.

R_1_, R_2_, L_1_, and L_2_ are attributed to the mass of the material, whereas R_3_ and C_3_ are attributed to its elastic properties. The resistors (R_1_ and R_2_) and inductors (L_1_ and L_2_) represent mechanical loss dissipation and mechanical energy storage within the piezoelectric layer, respectively. C_0_ and R_4_ denote the clamped capacitance and its associated dielectric loss. The factor α represents phase shift, while n(1 − α) defines the complex electromechanical transformer ratio [55]. This modified Mason formulation is well-suited for simulating transducers operating under complex external loading conditions.

**Figure 16:**
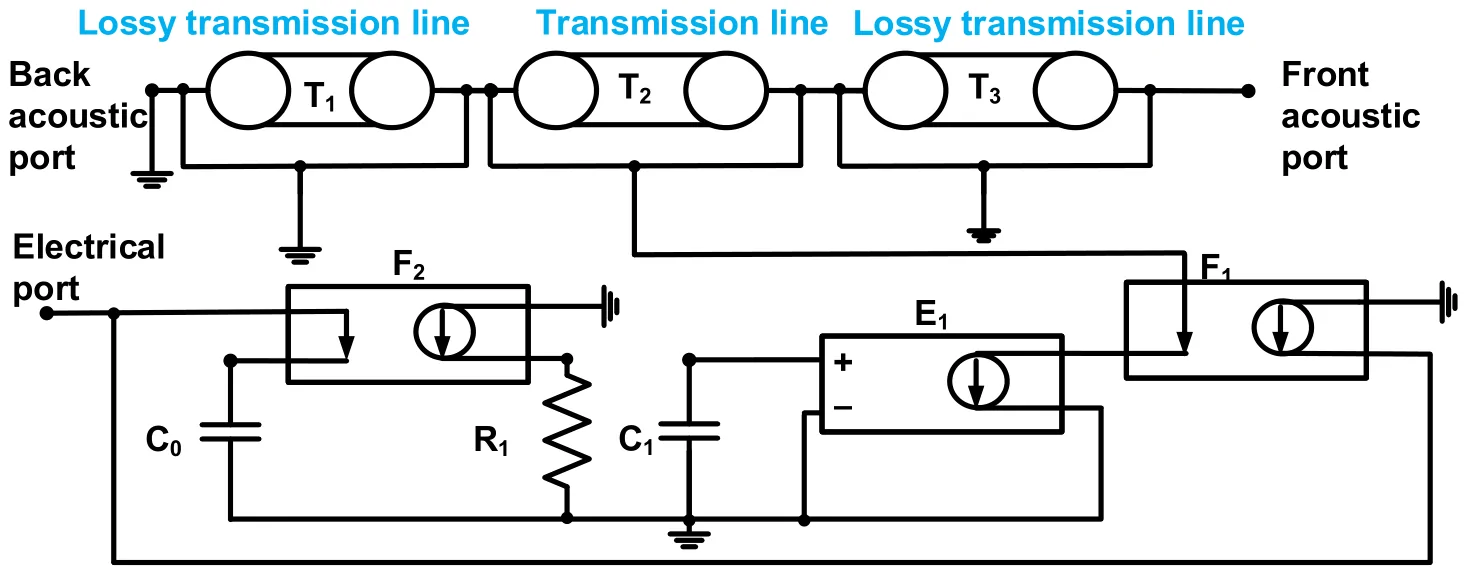
The conceptual equivalent circuit model based on the lossy transmission line and controlled source for the PVDF transducer. Reproduced from Ref. [56]. Copyright 2018, Elsevier.

Figure 16 shows the modified equivalent circuit model based on the controlled-source model and the lossy transmission line presented by Zheng et al. for the PVDF wideband piezoelectric transducer [56]. PVDF has a low mechanical quality factor, wide bandwidth, and low sensitivity. The frequency-dependent ideal transformer and negative clamped capacitances are replaced with a controlled source and a lossy transmission line.

Zheng et al. provided comparative data for an equivalent circuit model based on a lossy transmission line and a controlled source for a PVDF transducer. In the pulse–echo responses, the measured peak-to-peak echo amplitude was 0.1202 V_p-p_ [56]. The echo amplitudes obtained using the KLM model and the proposed model were 0.1103 V_p-p_ and 0.1295 V_p-p_, respectively [56]. In addition, the center frequencies of the measured echo, the KLM model, and the proposed model were 8.27 MHz, 8.4 MHz, and 8.27 MHz, respectively [56]. Therefore, the echo amplitude and center frequency predicted by the proposed model aligned more closely with the experimental data.

T_1_ represents the lossy transmission lines in the back acoustic port, and T_3_ represents the lossy transmission lines in the front acoustic port. E_1_, F_1_, and F_2_ are the controlled sources of the material parts, which represent the piezoelectric effects. The lossy transmission lines (T_1_ and T_3_) model the acoustic attenuation in the electrode and backing layers, respectively. The electrical parameters R_1_ and C_1_ in the controlled-source domain are extracted directly from impedance analyzer measurements. By eliminating both the transformer and negative clamped capacitance present in standard Mason/KLM models, frequency-dependent phase anomalies are minimized [56]. This controlled-source lossy transmission line model is especially useful for PVDF film transducers, effectively incorporating finite silver electrode thickness and backing wave velocity. In pulse–echo simulations, predicted peak-to-peak echo amplitudes using KLM, Mason, and this proposed model were 0.1103 V_p-p_, 0.1126 V_p-p_, and 0.1202 V_p-p_, compared to experimental results [56].

**Figure 17:**
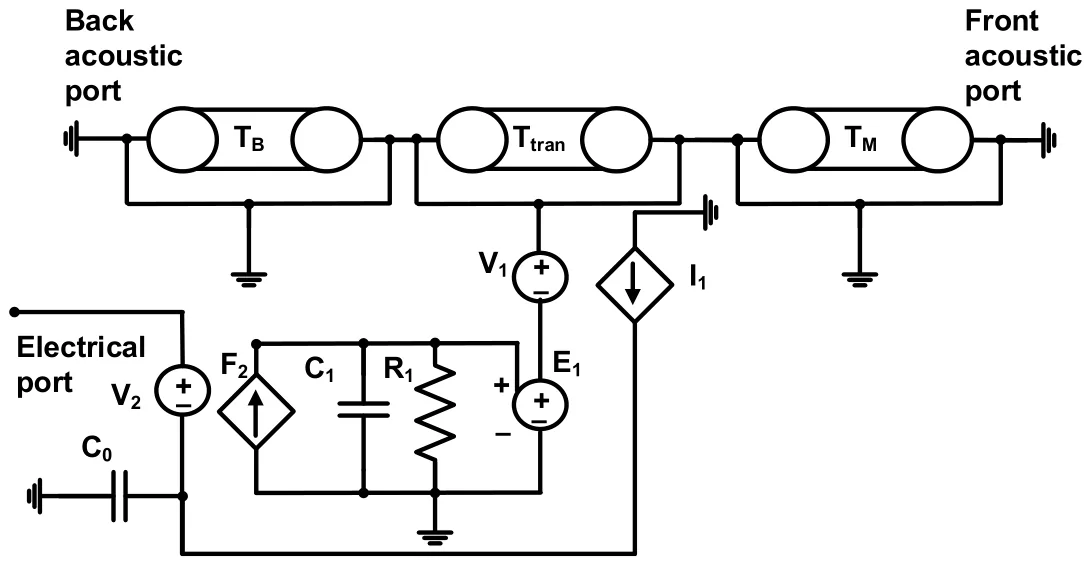
The equivalent circuit model using a lossy transmission line and controlled source with long-cable effects was presented by Shen et al. Adapted from Shen. Ref. [57] with permission under the terms of the CC BY 4.0 License, Copyright 2021, Frontier Media SA.

Figure 17 shows the equivalent circuit model based on a lossy transmission line and controlled sources, including cables, for high-frequency ultrasound transducer applications [57]. The presented model includes backing and matching layers, which are the core components of a high-frequency ultrasound transducer. The long cable effect is critically dependent on the performance of the high-frequency ultrasound transducer; therefore, a longer cable length can reduce the echo-signal amplitude [58]. This model does not require knowledge of mechanical properties such as matching and backing layers. Therefore, the derived model is useful for LTSPICE simulation.

T_B_ and T_M_ represent the lossy transmission lines for the backing and matching layers in the back and front acoustic ports, respectively. T_c_ and T_tran_ represent the coaxial cable with a lossy transmission line and piezoelectric material, respectively. Port E1 represents the electrical excitation source. The controlled sources were E_1_, I_1_, F_2_, and V_2_.

**Figure 18:**
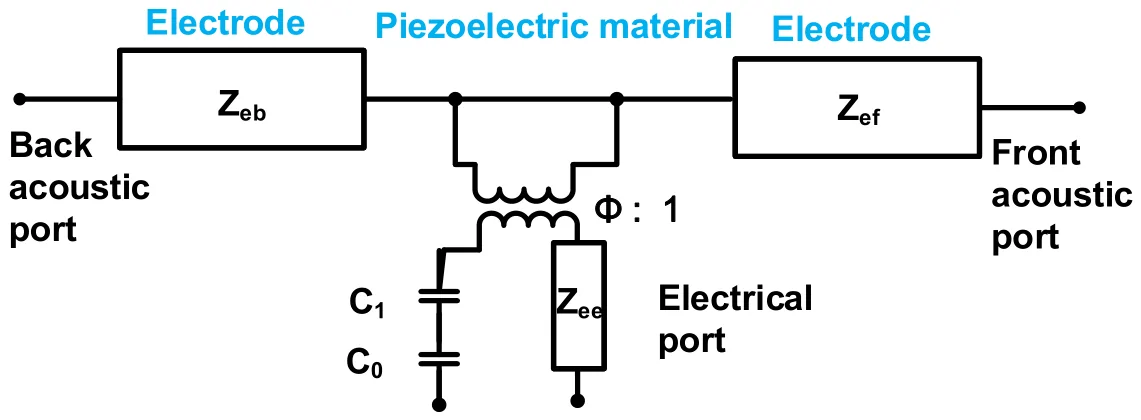
The conceptual equivalent circuit model using a piezoelectric material and electrodes. Redrawn from Ref. [59]. Z_eb_ and Z_ef_ represent the impedances at back and front acoustic ports, respectively. Z_ee_ represents the impedance at the electrical port.

As shown in Figure 18, a modified KLM model using silver electrodes and piezoelectric materials was proposed by Zawada et al. The proposed KLM model considers a 4 MHz high-intensity focused ultrasound (HIFU) with third and fifth harmonic components [59]. The clamped capacitance can be obtained as shown in Equation (32) [59]:

(32) $$C_{0}=\frac{\varepsilon_{0}\varepsilon^{S}\left(1-\mathrm{jtan}\delta \right)A}{d},$$

where ε_0_, ε^S^, tanδ, d, and A are the dielectric permittivity in air, the relative dielectric permittivity and loss factor of the piezoelectric material, and the thickness and surface area, respectively.

The ε^S^ can be obtained from Equation (33):

(33) $$\varepsilon^{S}=\frac{\varepsilon^{\sigma }}{1+k_{t}^{2}},$$

where ε^*σ*^ and k_t_ are the dielectric permittivities of the constant stress and the electromechanical coupling factor, respectively.

The second capacitance can be calculated as shown in Equation (34):

(34) $$C_{1}=-\frac{C_{0}}{k_{t}^{2}\mathrm{sinc}(\frac{w}{w_{0}})},$$

where w and w_0_ are the angular operating and anti-resonant frequencies.

The mechanical impedance of the piezoelectric material (Z_m_) can be obtained using Equation (35):

Z_m_ = Z_P_∙*A*, (35)

where Z_p_ and A denote the acoustic impedance and surface area of the piezoelectric material, respectively.

The electro-mechanical ratio (Φ) can be obtained using Equation (36) [59]:

(36) $$\Phi =k_{t}\sqrt{\left(\frac{\pi }{w_{0}C_{0}Z_{m}}\right)}\mathrm{sinc}(\frac{w}{w_{0}}),$$

In addition, the presented KLM model was verified using a fabricated transducer for radiation force balance measurement. For the first transducer, the simulated and measured center frequency were 11.76 MHz and 11.75 MHz, respectively, yielding a relative error of approximately 0.08% [59]. For the second transducer, the corresponding values were 11.13 MHz and 11.30 MHz, respectively, resulting in an error of approximately 1.5% [59]. The authors also reported that the magnitude error remained below 10 Ω.

**Figure 19:**
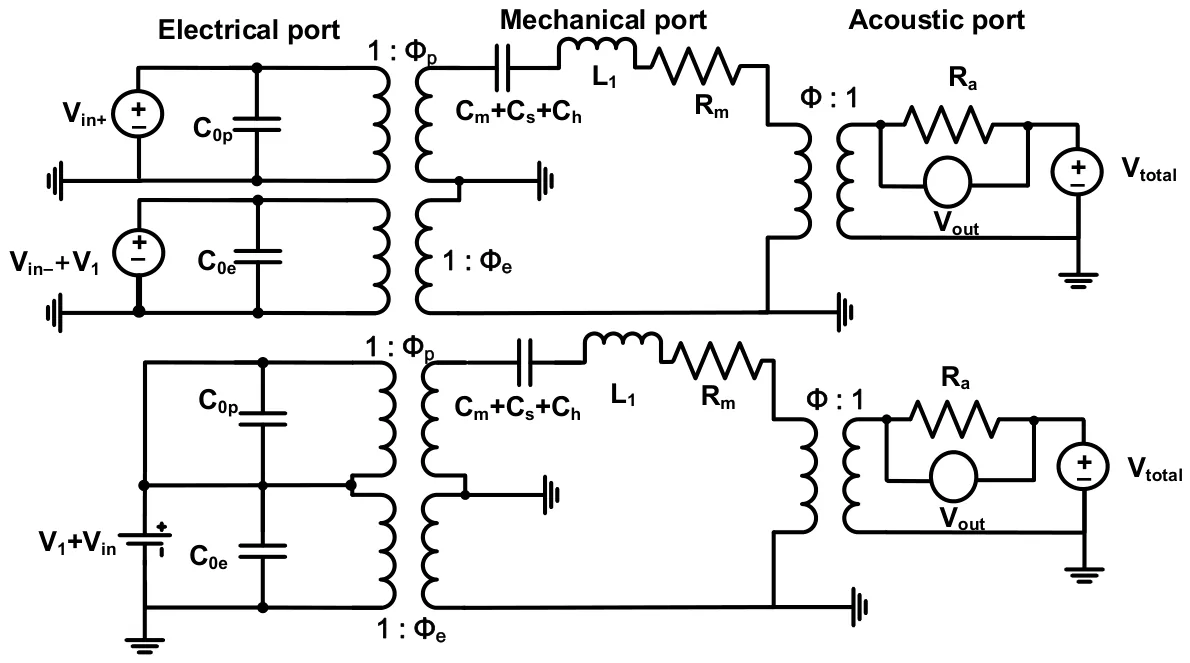
The simplified hybrid equivalent circuit model. Redrawn from Ref. [60].

Figure 19 shows the first hybrid equivalent circuit model composed of a piezoelectric micromachined ultrasonic transducer (PMUT) and a capacitive micromachined ultrasonic transducer (CMUT), which has dual force activation that has strain-induced activation and electrostatic force. The voltage-controlled source in the electrical port, the resistor–inductor–capacitor in the mechanical port, and the voltage and power sources in the acoustic port are shown. In the electrical port, three different excitation voltage sources (V_in+_, V_in-_, and V_in_) representing the excitation voltages in the top, bottom, and middle electrodes with a DC bias voltage (V_1_) were used. In addition, the clamp capacitances (C_op_ and C_oe_) and electromechanical turn ratios (Φ_p_ or Φ_e_) were used. In the mechanical port, a mechanical damper (R_m_), mechanical capacitance (C_m_), negative capacitance (C_s_) due to spring softening, and top electrode capacitance (C_h_) were used. In the acoustic port, the acoustic radiation area (Φ), acoustic impedance (R_a_), voltage source generated by the acoustic wave (P_in_), voltages reflecting transmitted acoustic pressure (V_out_), total acoustic power (V_total_), and effective acoustic radiation area (Φ) were used. This hybrid equivalent circuit model, based on the modified Mason Model, demonstrates the interaction in electrical, mechanical, and acoustic ports. Therefore, Wang et al. optimized each parameter to improve sensitivity and control bandwidth.

## 4. Discussion

**Figure 20:**
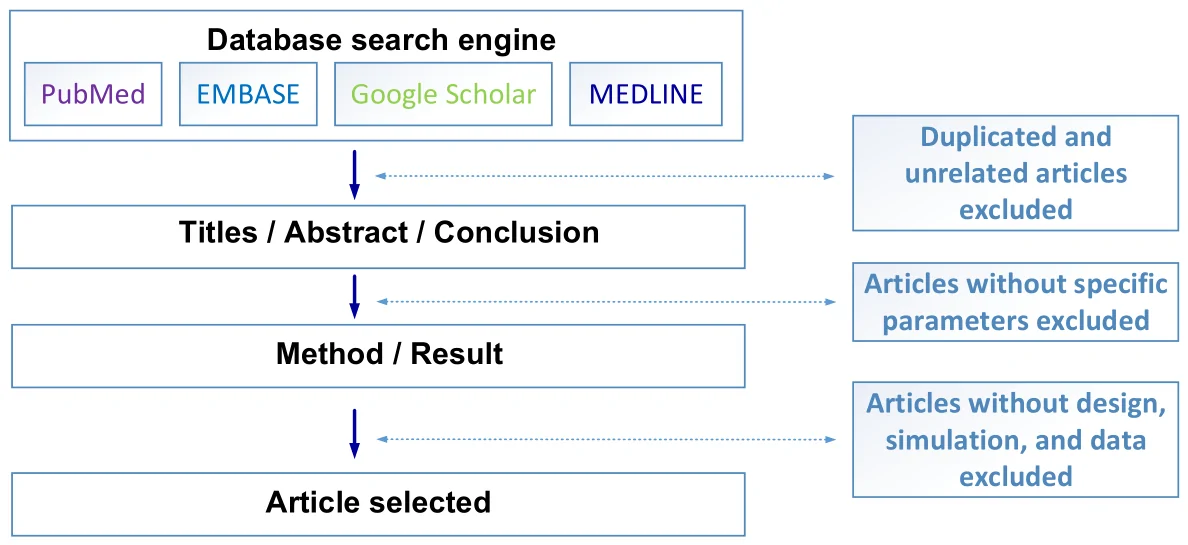
Article search flowchart for the equivalent circuit models.

First, articles related to the equivalent circuit models of piezoelectric transducers used for medical ultrasound applications published between 1970 and 2026 were collected using database search engines, including PubMed, EMBASE, Google Scholar, and MEDLINE. Various search terms were employed, such as equivalent circuit model, Krimholtz–Leedom–Matthaei, KLM, BVD, Mason, modified KLM, modified BVD, modified Mason, Butterworth van–Dyke, and their combinations. Second, studies unrelated to piezoelectric transducers, such as those on microwave-film bulk acoustic resonators, Tonpilz transducers, and general impedance-matching techniques, were carefully screened to select studies centered on equivalent circuit models for piezoelectric transducers. Finally, the abstract, introduction, method, and conclusion sections were reviewed to determine the equivalent circuit model and analyze piezoelectric transducers. Subsequently, the selected articles were studied and analyzed in this review. Figure 20 illustrates the literature search and screening flowchart.

The major piezoelectric materials used in the transducers for medical ultrasound applications are PZT-5H, single crystals, and PVDF [24]. Some equivalent circuit models have been used for simulation verification using such piezoelectric materials, or to run the equivalent circuit model in PSPICE or LTSPICE programs before system integration. Equivalent circuit models have been presented for the straightforward analysis of piezoelectric transducer designs and the impedance-matching circuits used in ultrasound systems. The first Mason model has a frequency-dependent ideal transformer and negative-clamped capacitance. In the classical Mason model, the negative clamped capacitance (–C_0_) arises as a physical and mathematical consequence of modeling bidirectional electromechanical coupling, acting to cancel reaction stress induced by mechanical deformation within the electrical domain [35]. Simply omitting this negative capacitance eliminates convergence issues in SPICE-based ASIC simulators; however, doing so introduces substantial errors when predicting the electromechanical coupling coefficient (k_t_), as well as resonant/anti-resonant frequencies in high-coupling transducers [27,38]. To eliminate simulation instability without sacrificing accuracy, voltage-controlled sources were introduced [38,61]. Mathematically, this substitution is exact and fully equivalent to the original formulation. Nevertheless, a clear trade-off exists: controlled-source implementations can induce numerical convergence difficulties during transient PSPICE simulations and increase network complexity when high-frequency parasitic components are included. Other modified Mason models include dielectric, elastic, and piezoelectric losses to estimate the piezoelectric transducer model more accurately when using different external load configurations [55]. A modified Mason model was also proposed as a hybrid model for the CMUT and PMUT to improve the gain and bandwidth of the pulse–echo response [60].

In addition, a lossy transmission line and controlled sources were proposed to estimate a more accurate model for the PVDF transducer, which considers the properties of the silver electrodes and backing materials [56]. Controlled sources and lossy transmission lines with long cable models have been proposed for high-frequency ultrasound transducer applications [57]. The presented black-box model does not require knowledge of specific mechanical parameters, and the model component data were obtained from the measured impedance and echo signals from the hydrophone. Therefore, this model could be useful in MATLAB simulation tools, such as Simulink. The modified KLM model has a lossy transmission line that is useful for considering mechanical loss; therefore, the internal model in the PSPICE program can be used [40]. A modified transmission line was also used to analyze the effects of the electrical terminal load on a multilayered piezoelectric transducer. A modified KLM model for the HIFU transducer with the third and fifth harmonic components was proposed for the thickness mode. In the equivalent circuit model, piezoelectric materials and electrodes were included in the analysis.

A modified BVD model was introduced to estimate the energy dissipation caused by the loss of piezoelectric materials [41]. Other modified BVD models have been presented for multiple PZT material structures or multiple resonant frequency points for broadband impedance-matching network capabilities [49,50]. Another modified BVD model with mechanical and electrical losses was useful for high-frequency ultrasound imaging applications.

Table 1 summarizes the characteristics, contributions, and applications of the piezoelectric transducers presented to date for medical ultrasound applications. Therefore, academic ultrasound researchers and circuit/system designers can select the types of equivalent circuit models that are useful for the respective performance specifications.

Electrical impedance-matching circuits are one of the applications of the equivalent circuit models of piezoelectric transducers. Using the BVD models, electrical matching circuits have been utilized for high-impedance ultrasonic transducers, nondestructive testing, microelectronic bonding, and broadband ultrasound transducer applications [62,63,64]. Another application of equivalent circuit models is the pulse–echo system evaluation [65]. To improve the performance of high-frequency piezoelectric transducers, equivalent circuit models, such as KLM models, have been utilized [66]. Using the BVD model, a high-power acoustic signal could be produced from a power amplifier, or the echo signal from the preamplifier could be improved in order to transmit an optimal sinusoidal wave from the transmitter [67,68,69]. In addition, an impedance spectroscopy measurement circuit was used based on the BVD model [70].

After series-to-parallel or parallel-to-series impedance conversion, the BVD model can be converted into one with a series or parallel resistance and capacitance [71,72]. In some studies on electrical impedance-matching circuits or ultrasound system development, impedance models with resistances and capacitances have been used. Such an impedance model can be obtained from the fabricated piezoelectric transducer in an electrical impedance analyzer. This model has been used for high-frequency ultrasound transducer fabrication, cell trapping, and pulse–echo stimulator applications [73,74,75]. Therefore, we conclude that accurate equivalent circuit models can be useful for improving the performance of piezoelectric transducers, ultrasound systems, and other medical applications.

For circuit design or transducer fabrication researchers, the table and flowchart are provided when selecting the equivalent circuit model for piezoelectric transducer applications. The specific parameters are selected from the previous articles. To provide guidelines for selecting the equivalent circuit model with specific application parameters, Table 2 is shown below.

**Figure 21:**
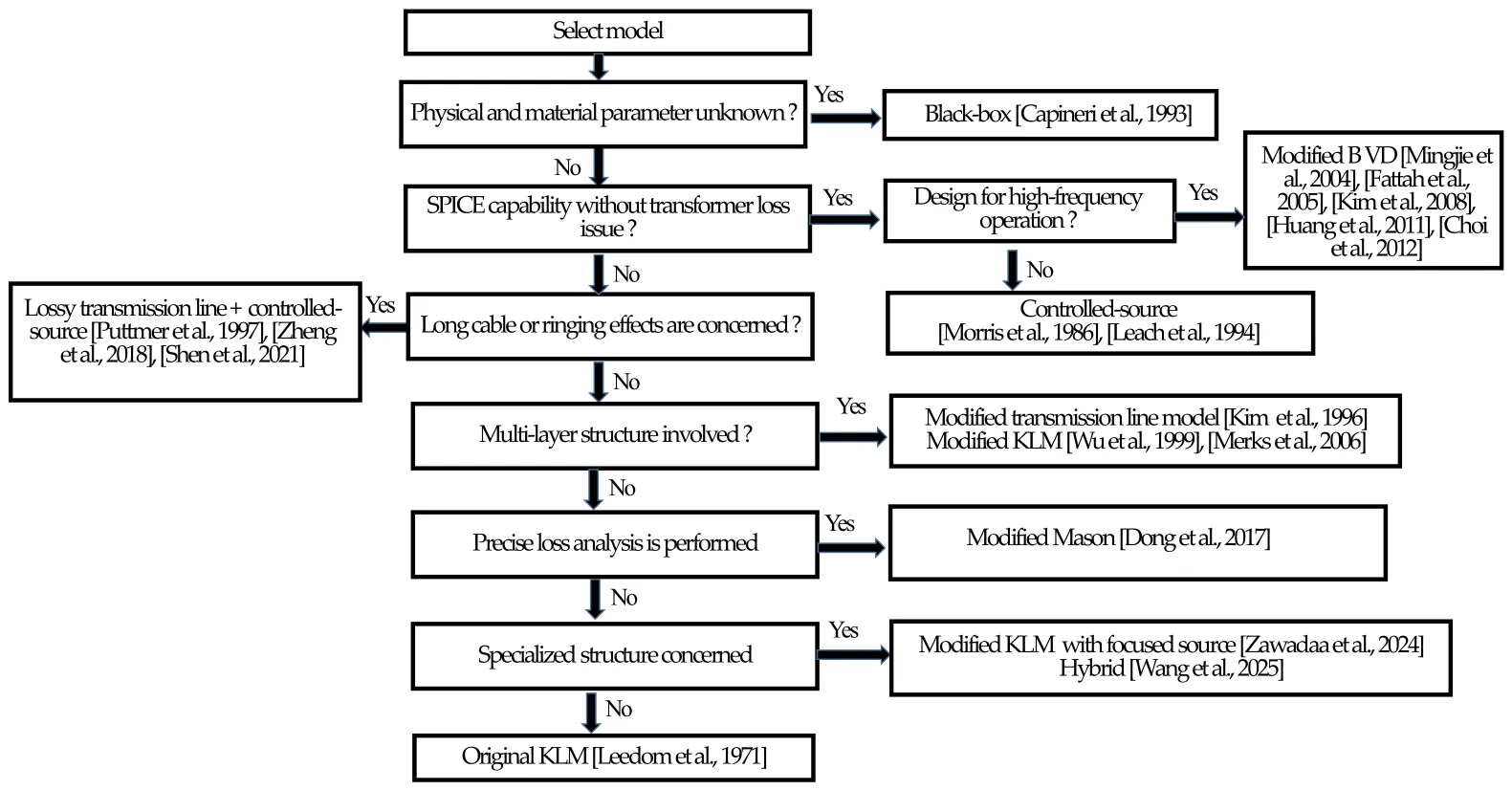
Flowchart of specific constraints for the equivalent circuit model, including black-box [37], controlled-source [35,38], modified BVD [43,44,49,50,54], modified transmission line [39] and KLM [41,47], modified Mason [55], modified KLM with focused source [59], hybrid [60], lossy transmission line + controlled source [40,56,57], and the original KLM models [34].

Figure 21 shows a flowchart of the specific constraints for the equivalent circuit model.

## 5. Conclusions

Several review articles have previously addressed piezoelectric transducers from various perspectives, such as functional materials, structural design, fabrication techniques, and impedance matching. For instance, Rathod reviewed electrical impedance-matching techniques for piezoelectric sensors and actuators [76], as well as acoustic impedance-matching strategies [77]. Zhou et al. summarized piezoelectric single-crystal ultrasonic transducers and their fabrication methods [12], while Shung et al. reviewed high-frequency piezoelectric materials for medical ultrasound [78]. Additionally, Wang et al. surveyed analytical models specifically for collapse-mode capacitive micromachined ultrasonic transducers (CMUTs) [79].

While these previous reviews focused primarily on material selection, fabrication steps, or external matching networks, the present review provides a comprehensive taxonomy, physical interpretation, and quantitative comparison specifically dedicated to equivalent circuit models (Mason, KLM, BVD, and modified variants). To the best of our knowledge, a dedicated review focused on model selection criteria and systematic equivalence mapping for medical ultrasound transducers has not been previously reported.

The piezoelectric transducers must be accurately modeled to achieve efficient power transfer. If efficient power transfer is achieved with relatively low power consumption, the variation in electrical impedance due to additional heat can be reduced. Therefore, considering the losses and mismatched electrical impedances caused by the use of improper equivalent circuit models is critical for performance, and an accurate model of the piezoelectric transducer could be helpful in the design.

The specific parameters are provided for piezoelectric transducer selection. This review article provides design guidelines for optimizing the performance of piezoelectric transducers, ultrasound circuits, and systems. Therefore, in system development, an accurate model of the piezoelectric transducer can be helpful before mechanical transducer fabrication or the development of electrical impedance-matching networks.

## Figures and Tables

**Table 1:** Summary of the equivalent circuit models of the piezoelectric transducers for medical ultrasound applications.

Articles	Model Characteristics	Contribution
[34]	The original KLM model	Equivalent circuit model including a frequency-dependent ideal transformer and transmission line for one-dimensional multi-layer analysis.
[35]	Modified Mason’s model using the source	The negative clamp capacitance and ideal transformer in Mason’s model were replaced with voltage and current sources so the model could be used in the SPICE program. The matching and backing layers were implemented with transmission line and lumped circuit elements.
[37]	Black-box model	The model components were obtained from the impedance and echo signals. This can be used in the MATLAB simulation tools because it is an analytical model in the Laplace domain. Therefore, it helps minimize mismatch between the signal generator and transducer.
[38]	The original controlled-source-based model	Controlled sources replaced the ideal transformer in the thickness-mode transducer. It is useful for SPICE simulation because displacement, velocity, force, and pressure components are replaced by the controlled sources.
[39]	Modified transmission line in the equivalent circuit model	Useful for analyzing the effects of the electrical terminal load on multi-layered piezoelectric transducers. It helps analyze the pulse duration and echo waveform ringing effects in the time domain because this model can describe the impedance of the pulse generator and receiver.
[40]	Lossy transmission line and controlled source	The internal model used a lossy transmission line to represent mechanical losses in the PSPICE program. The peak magnitude, quality factor, and bandwidth values are well matched in the high-power generation because the dielectric, elastic, mechanical, and piezoelectric losses are mapped with circuit elements in the PSPICE model. It also helps calculate the ringing attenuation time for a lossy piezoceramic transducer.
[41]	Modified KLM model	A lossy acoustic transmission line and matching layer were used for the piezoelectric transducer with low loss and wide bandwidth. Therefore, this model can be integrated with the parametric sweep function of the transient and AC analysis in the PSPICE program.
[43]	Modified BVD model	Useful for estimating energy dissipation caused by material loss in multiple higher-order resonance modes with harmonics for piezoceramic transducers.
[44]	Modified BVD model	Considers the higher vibration modes for PZT-5H material with high mechanical quality factor. In the model, additional radiation resistance, which represents the acoustic load, was added.
[47]	Modified KLM model	Valid for multi-layer structures such as PVDF layer and PZT-2 elements in the bonding layers, cable, and protective coating for bladder volume measurement applications.
[49]	Modified BVD model	Used for multiple resonance and anti-resonance points in loaded piezoelectric ceramic materials. Therefore, the magnitude and phases in more accurate electrical modeling were achieved.
[50]	Modified BVD model	Has multiple resonant frequencies for broadband impedance matching of piezoelectric transducers. Based on the measured admittance data, the parameters were extracted to reflect the non-ideal behaviors caused by the fabrication and crack issues. Therefore, this helps improve the power and signal-to-noise ratio.
[54]	Modified BVD model	Considers the mechanical and electrical losses in high-frequency medical imaging applications. This model considers the parasitic impedances, so the impedance characteristics at the resonant and anti-resonant frequencies are more accurate than the conventional BVD model in high-frequency piezoelectric materials.
[55]	Modified Mason’s model	The model takes into account the dielectric, elastic, and piezoelectric losses, including the external loads. Therefore, it helps model the piezoelectric transducer with a high quality factor for high-power environments.
[56]	Lossy transmission line and controlled source	Using controlled sources and a lossy transmission line, which represent the piezoelectric effect and acoustic wave propagation, the presented model can remove the frequency-dependent ideal transformer and negative clamped capacitances in PVDF piezoelectric transducers.
[57]	Lossy transmission line and controlled source with cable model	Because controlled sources and lossy transmission lines with long cable effects are considered, this model is useful for high-frequency ultrasound transducers.
[59]	Modified KLM model with focused source	The modified KLM model, assuming silver electrodes and piezoelectric material, was proposed to consider the third and fifth harmonic components for a focused-type transducer.
[60]	Hybrid model	The controlled source and transformer in the equivalent circuit model based on the Mason model were used for the hybrid PUMT and CMUT.

**Table 2:** The specific parameter summary of the equivalent circuit models.

Articles	Model	SPICE Compatibility	Multi-Layer Structure	Losses	Cable Effects	Empirical Fitting	High-Frequency Operation
[34]	Original KLM	△	○	△	×	×	×
[35]	Modified Mason	△	×	△	×	×	×
[37]	Black-box	△	×	×	×	◎	×
[38]	Original controlled source	○	×	△	×	×	×
[39]	Modified transmission	○	◎	○	○	×	×
[40,56]	Lossy transmission + Controlled source	○	◎	○	◎	△	△
[57]	Lossy transmission + Controlled source + Cable	○	◎	◎	◎	△	△
[41,47]	Modified KLM	○	◎	○	×	×	×
[43,44,49,50,54]	Modified BVD	◎	×	○	△	○	○
[55]	Modified Mason	△	×	◎	△	×	×
[59,60]	Specialized (Hybrid, Modified KLM with focused source)	△	◎	○	△	△	◎

◎: Recommended, ○: Acceptable, △: Limited, ×: Poor/Not supported.

## Data Availability

Data are contained within the article.

## Abbreviations

FEM	Finite-element model
KLM	Krimholtz–Leedom–Matthaei
BVD	Butterworth–Van Dyke
ASIC	Application-specific integrated circuit
PZT	Lead zirconate titanate
PVDF	Polyvinylidene fluoride
HIFU	High-intensity focused ultrasound
CMUT	Capacitive micromachined ultrasonic transducer
PMUT	Piezoelectric micromachined ultrasonic transducer
